# Pasireotide‐induced hyperglycemia in a patient with Cushing's disease: Potential use of sodium‐glucose cotransporter 2 inhibitor and glucagon‐like peptide‐1 receptor agonist for treatment

**DOI:** 10.1002/ccr3.3230

**Published:** 2020-08-13

**Authors:** Masato Shikata, Kenji Ashida, Yuka Goto, Ayako Nagayama, Shimpei Iwata, Mamiko Yano, Nao Hasuzawa, Kento Hara, Kazutoshi Mawatari, Kiyohiko Sakata, Munehisa Tsuruta, Nobuhiko Wada, Masatoshi Nomura

**Affiliations:** ^1^ Division of Endocrinology and Metabolism Department of Internal Medicine Kurume University School of Medicine Fukuoka Japan; ^2^ Division of Cardiovascular Medicine Department of Internal Medicine Kurume University School of Medicine Fukuoka Japan; ^3^ Department of Neurosurgery Kurume University School of Medicine Fukuoka Japan

**Keywords:** Cushing's disease, diabetes mellitus, glucagon‐like peptide‐1 receptor agonist, pasireotide, sodium‐glucose cotransporter 2 inhibitor

## Abstract

Pasireotide improves hypercortisolemia and induces hyperglycemia via somatostatin receptor type‐5 stimulation. GLP‐1RA and SGLT2 inhibitor potentially help regulate hyperglycemia in patients with Cushing's disease, especially after pasireotide administration.

## Graphical abstract

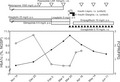

## 1 INTRODUCTION

Pasireotide, a somatostatin receptor analog, is a promising drug for Cushing's disease.^1^ The broad stimulation of somatostatin receptor subtypes, including somatostatin receptor type‐5 (SSTR5), suppresses adrenocorticotropic hormone (ACTH) levels, along with hypercortisolemia, and reduces tumor volume.^2^ However, SSTR5 stimulation also suppresses insulin secretion from pancreatic β‐cells, leading to hyperglycemia.^3^, ^4^, ^5^ Owing to this inevitable adverse event, physicians hesitate to prescribe the beneficial drugs to relevant patients.

A certain population of patients with Cushing's disease presents with refractory disease,^6^ because the underlying ACTH producing‐tumor is sometimes difficult to identify and/or completely resect. Therefore, persistent hypercortisolemia frequently impairs glucose metabolism in patients with Cushing's disease. Various drugs have been reported as therapeutic alternatives in hypercortisolemia, particularly in patients with Cushing's disease.^7^

However, optimal methods of managing hyperglycemia in patients having Cushing's disease and receiving pasireotide remain unclear, despite the reported association between impaired glucose metabolism and pasireotide administration.^8^, ^9^ Here, we describe a case of Cushing's disease with pasireotide‐induced hyperglycemia, which was managed with glucagon‐like peptide‐1 receptor agonist (GLP‐1RA) and sodium‐glucose cotransporter 2 (SGLT2) inhibitor. Improvement of insulin secretion and sensitivity was observed over a 6‐month clinical course. The study shows that GLP‐1RA and SGLT2 inhibitor have the potential to manage exacerbated hyperglycemia due to pasireotide administration in patients with Cushing's disease.

## 2 CASE HISTORY/EXAMINATION

**FIGURE 1:**
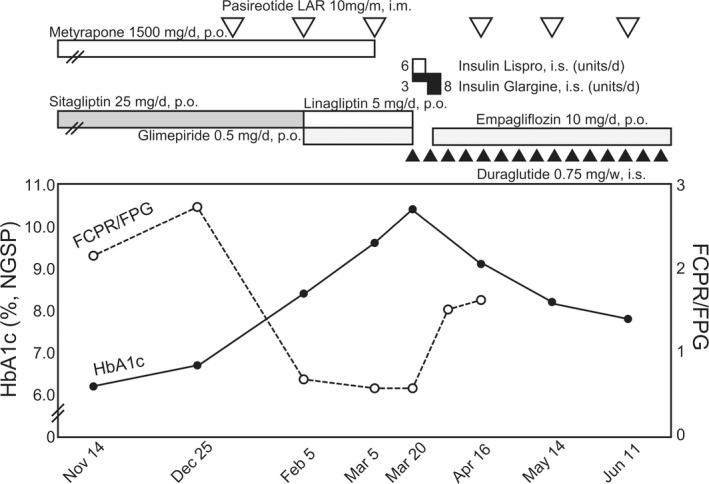
Clinical course of the present case. Pasireotide LAR administration, followed by elevation of HbA1c levels and suppression of FCPR/FPG, representing insulin secretion. Subcutaneous and transient injection of insulin up to 9 units/d (insulin lispro 2 units before every meal [3 times/d] and insulin glargine 3 units at night). After initiation of dulaglutide, and subsequently empagliflozin, insulin injection could be tapered and finally discontinued. Dulaglutide and empagliflozin administration improved both FCPR/FPG and HbA1c values. FCPR/FPG, FCPR × 100/FPG; FCPR, fasting C‐peptide (ng/mL); FPG, fasting plasma glucose (mg/dL); HbA1c, hemoglobin A1c; LAR, long acting repeatable

A 64‐year‐old Japanese man was admitted to Kurume University Hospital on March 19, 2019, owing to fatigue and hyperglycemia. He had been diagnosed with Cushing's disease 7 years ago and had undergone endoscopic endonasal trans‐sphenoidal surgery in July 2012. However, ACTH and cortisol levels remained high; hence, a residual tumor was suspected. He had been diagnosed with diabetes mellitus 2 years prior to the diagnosis of Cushing's disease. Upon initiation of metyrapone (1250 mg/d) in January 2013, both ACTH and cortisol levels were normalized, and hemoglobin A1c (HbA1c) levels (NGSP: National Glycohemoglobin Standardization Program) improved to approximately 6.0%. However, Cushing's disease was exacerbated with fatigue in July 2018 (ACTH level, 49.3 pg/mL; Serum cortisol level, 21.3 μg/dL; urinary free‐cortisol level, 81.2 μg/d), and 10 mg/mo of pasireotide was initiated on January 8, 2019, 2 months prior to hospital admission. After pasireotide therapy, his HbA1c increased to 8.4% in February and 9.6% in March 2019. However, administration of metformin and dipeptidyl peptidase‐4 (DPP4) inhibitor failed to improve his hyperglycemia. The clinical course is presented in Figure 1.

**FIGURE 2:**
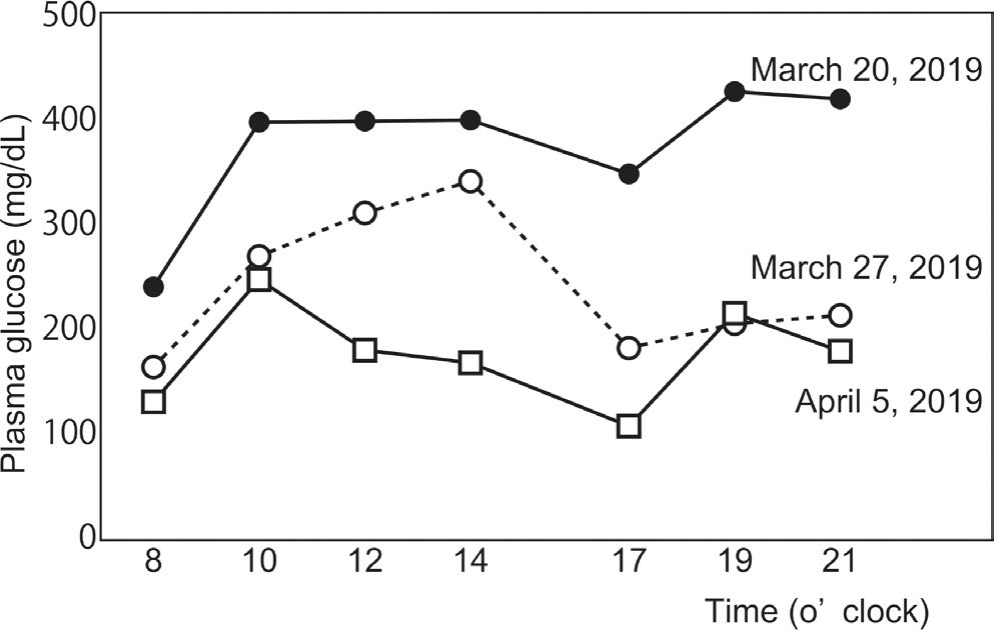
Daily plasma glucose profile during the clinical course. Plasma glucose profile was determined upon admission. Closed circle with line (March 20, 2019), insulin lispro (2 units before every meal), and insulin glargine (3 units before dinner); open circle with broken line (March 27, 2019), GLP‐1RA (0.75 mg/wk) and insulin glargine (8 units before dinner); open square with line (April 5, 2019), GLP‐1RA (0.75 mg/wk) and SGLT2 inhibitor (10 mg empagliflozin). GLP‐1RA administration improved the glucose profile, and SGLT2 inhibitor administration improved the plasma glucose profile. GLP‐1RA, glucagon‐like peptide‐1 receptor agonist

Physical examination revealed Cushingoid features, including central obesity and a full moon‐like face. He had a height of 160 cm, weight of 56 kg, and no noted edema. Laboratory examination upon admission (Table 1) revealed a high HbA1c level of 10.4% and hyperglycemia of 242 mg/dL. Administration of pasireotide (10 mg/mo) reduced plasma ACTH, serum cortisol, urinary free‐cortisol, and dehydroepiandrosterone‐sulfate levels to 45.6 pg/mL, 10.8 μg/dL, 59.9 μg/d, and 50 μg/dL, respectively (Figure 1 and Table 1). On the contrary, fasting plasma glucose and HbA1c levels increased in accordance with the disruption of endogenous insulin secretion (Table 2). Multiple daily insulin injections, up to 9 units/d, were initiated on March 20, 2019, because hyperglycemia was observed. Additionally, subcutaneous administration of 0.75 mg of dulaglutide once a week was initiated on March 22, 2019 for further therapy to improve hyperglycemia. Hyperglycemia was improved and insulin could be tapered to 8 units/d, 4 days after the initiation of dulaglutide (March 26, 2019) (Figure 1). Daily plasma glucose profile, 5 days after the initiation of dulaglutide (March 27, 2019), is shown in Figure 2. Furthermore, 10 mg/d of empagliflozin was administered after another 2 days (March 28, 2019), following which the daily plasma glucose profile improved rapidly and insulin could be discontinued on April 1, 2019 (Figure 2). After 3 months, hypercortisolemia and glucose impairment in the patient were well‐regulated, and his health improved owing to an overall improvement in hyperglycemia at every visit to our outpatient center (Figure 1).

**TABLE 1:** Laboratory data at admission

Parameters	Value	Parameters	Value
Complete blood cell count		Serum chemistry	
Red blood cell count, ×10^4^/µL	338	Aspartate aminotransferase, U/L	34
Hemoglobin, g/dL	9.2	Alanine aminotransferase, U/L	24
Hematocrit, %	30.3	γ‐glutamyltransferase, U/L	393
White blood cell count, /µL	4200	Albumin, g/dL	2.8
Neutrophil, %	66.1	Creatine kinase, U/L	51
Eosinophil, %	2.1	Triglyceride, mg/dL	104
Lymphocyte, %	23.9	LDL‐C, mg/dL	74
Platelet, ×10^4^/µL	16.5	Blood urea nitrogen, mg/dL	22
Endocrinology		Creatinine, mg/dL	1.01
Adrenocorticotropic hormone, pg/mL	45.6	Sodium, mmol/L	138
Cortisol, µg/dL	10.8	Potassium, mmol/L	4.3
Dehydroepiandrosterone‐sulfate, µg/dL	50	Chloride, mmol/L	105
Growth hormone, ng/mL	0.15	Calcium, mg/dL	9.6
Insulin‐like growth factor‐1, ng/mL	19	Phosphate, mg/dL	2.9
Prolactin, ng/mL	9.3	C‐reactive protein, mg/dL	0.47
Thyroid‐stimulating hormone, µIU/mL	2.45	Glucose metabolism	
Free thyroxine, ng/dL	1.53	Plasma glucose, mg/dL	242
Luteinizing hormone, mIU/mL	14.5	HbA1C, % (NGSP)	10.4
Follicular stimulating hormone, mIU/mL	21.5	Immunoreactive insulin, µU/mL	2.0

Abbreviations: HbA1c, hemoglobin A1c; LDL‐C, low‐density lipoprotein cholesterol; NGSP, National Glycohemoglobin Standardization Program.

**TABLE 2:** Changes in diabetes‐related parameters upon combinatorial administration of pasireotide, GLP‐1RA, and SGLT2 inhibitor

	Date	Nov 14/2018	Dec 3/2018	Mar 20/2019	Apr 3/2019
Treatment					
Pasireotide (weeks from start of administration)		‐	‐	+ (10 wk)	+ (12 wk)
GLP‐1RA (weeks from start of administration)‐		‐	‐	‐	+ (2 wk)
SGLT2 inhibitor (weeks from start of administration)‐		‐	‐	‐	+ (1 wk)
Diet tolerance test (75 g carbohydrate, 60% of total diet calorie)					
Plasma glucose, mg/dL	0 min	93	132	242	139
	60 min	196	210	368	204
	120 min	219	186	410	233
Serum C‐peptide, ng/mL	0 min	2.0	3.6	1.4	2.1
	60 min	4.6	5.8	2.4	2.8
	120 min	8.4	10.0	3.9	5.8

Abbreviations: GLP‐1RA, glucagon‐like peptide‐1 receptor agonist; SGLT2 inhibitor, sodium‐glucose cotransporter 2 inhibitor.

## 3 DISCUSSION

This is the first report of a case with Cushing's disease complicated with diabetes mellitus, which was exaggerated by pasireotide treatment and eventually ameliorated with GLP‐1RA and SGLT2 inhibitor. The prognosis of patients with refractory Cushing's disease may be improved by reduction of hypercortisolemia by management of the complicating hyperglycemia using antidiabetic therapy.

Pasireotide‐induced insulin suppression led to hyperglycemia, which was attenuated by GLP‐1RA, although not by a DPP4 inhibitor. Pasireotide ameliorated hypercortisolemia through the stimulation of SSTR5, thereby, suppressing the secretion of ACTH.^1^, ^2^, ^4^ However, pasireotide also suppressed the secretion of insulin^3^, ^4^, ^5^, ^9^ directly via SSTR5 in pancreatic β cells and indirectly by suppressing GIP and GLP1.^3^, ^5^, ^9^ Insulin,^4^ metformin,^4^, ^5^ and DPP4 inhibitors^5^ are alternative candidate drugs for pasireotide‐induced hyperglycemia. However, in this study, neither low‐dose metformin nor DPP4 inhibitor could improve the impaired glucose metabolism (Figure 1). Notably, the administration of GLP‐1RA showed potential to improve the secretion of insulin (Table 2 and Figure 1) and enhanced the metabolism of glucose. Furthermore, considering the suppression of GLP1 by the administration of glucocorticoid^10^ and pasireotide,^3^, ^9^ GLP‐1RA may be considered a suitable alternative for improving impaired glucose metabolism in individuals having hypercortisolemia and receiving pasireotide.

SGLT2 inhibitor showed potential to improve the plasma glucose profile after the administration of GLP‐1RA. Its administration increased the levels of urinary glucose and improved the plasma glucose profile (Figure 2).^11^ Because glucocorticoids increase urinary glucose excretion by reducing the glucose threshold levels in excretion,^12^, ^13^ pasireotide might exert a diabetic effect by reducing urinary glucose and ameliorating hypercortisolemia. Therefore, the SGLT2 inhibitor may effectively exert anti‐diabetic effects via reverse elevation of urinary glucose excretion. The SGLT2 inhibitor improved insulin sensitivity in skeletal muscles and visceral adipose tissue via ectopic fat reduction,^14^, ^15^ whereas glucocorticoid caused the accumulation of ectopic fat, particularly in adipose tissues and skeletal muscles,^16^ leading to insulin resistance.^17^ Furthermore, pasireotide has been shown to reduce glucagon secretion from pancreatic α‐cells.^5^, ^9^ Low levels of serum glucagon exacerbate the fluctuation of daily glucose profiles, especially during hypoglycemia.^18^ In this context, SGLT2 inhibitors may help stabilize daily plasma glucose profiles, via the recovery of suppressed glucagon secretion.^19^

Control of pasireotide‐induced hyperglycemia may help prevent hypoglycemia and cardiovascular events and enhance the prognosis of patients with refractory Cushing's disease. Both GLP‐1RA and SGLT2 inhibitor have potential to exert beneficial effects, such as prevention of cardiovascular events, as well as regulation of glucose metabolism.^20^, ^21^ In this context, both drugs should be considered as alternatives for administration to patients with Cushing's syndrome and diabetes mellitus, along with pasireotide, to reduce the risk of cardiovascular events. Although the administration of SGLT2 inhibitor may be contraindicated in patients with reduced insulin secretion to prevent euglycemic ketoacidosis, GLP‐1RA‐enhanced insulin excretion may provide a safety margin for treatment with an SGLT2 inhibitor in patients with pasireotide‐induced hyperglycemia.

The present study has some limitations. Owing to the small study size, we could not decisively conclude whether GLP‐1RA and SGLT2 inhibitor could be beneficial drugs for treating pasireotide‐induced hyperglycemia in patients with Cushing's disease. Large‐sized studies in future may support our findings. Additionally, we could not use high‐dose metformin, owing to chances of severe congestive heart failure, although metformin would be the first recommended drug for this condition.^22^

In conclusion, this study reports the case of a patient with intractable Cushing's disease and hyperglycemia, along with reduction of endogenous insulin secretion by pasireotide administration. GLP‐1RA and SGLT2 inhibitor showed potential to improve glucose metabolism in this condition. Improvement of prognosis of patients with refractory Cushing's disease is expected to be demonstrated using this regimen, thereby, preventing and improving hypercortisolemia‐related complications and pasireotide‐induced hyperglycemia.

## CONFLICT OF INTEREST

None declared.

## AUTHOR CONTRIBUTIONS

MS: involved in study design, data collection, drafting, interpretation of data, and revision. KA: involved in study design, data collection, drafting, interpretation of the data, review, and revision. YG: involved in data collection, interpretation of the data, and review. AN: involved in interpretation of the data and review. SI: involved in interpretation of the data and review. MY: involved in interpretation of the data and review. NH: involved in interpretation of the data and review. KH: involved in interpretation of the data and review. KM: involved in data collection, interpretation of the data and review. KS: involved in data collection, interpretation of the data and review. MT: involved in interpretation of the data and review. NW: involved in interpretation of the data and review. MN: involved in study design, drafting, interpretation of the data, review and revision. All authors provided inputs for preparation of the manuscript and have read and approved the final version for submission.

## ETHICAL APPROVAL

All procedures complied with the ethical standards of the Institutional Review Board of the Kurume University School of Medicine and the 2013 Declaration of Helsinki. This report was approved by the Ethics Committee of Kurume University Hospital (2019‐080).
